# Influence of Solvent Relative Permittivity in Swab Spray Mass Spectrometry

**DOI:** 10.3390/molecules29174274

**Published:** 2024-09-09

**Authors:** Thomas Michael Muggli, Stefan Schürch

**Affiliations:** Department of Chemistry, Biochemistry and Pharmaceutical Sciences, University of Bern, 3012 Bern, Switzerland; thomas.muggli@unibe.ch

**Keywords:** swab spray mass spectrometry, electrospray ionization, Taylor cone formation, ambient ionization, relative permittivity, paper spray

## Abstract

The influence of solvent properties on ion generation by swab spray ionization was investigated. The ability of a variety of solvents of different relative permittivity, surface tension, and viscosity to form a stable and reproducible electrospray was examined. It is demonstrated that in swab spray ionization, a crucial balance between solvent composition, applied potential, and the solvent flow fed to the swab head must be maintained. The solvent composition was found to significantly affect the shape of the Taylor cone and the emerging cone jet, which eventually have an impact on the resulting ion yield. The results indicate that the relative permittivity of solvents measured under standard conditions is the main factor governing jet shaping, and consequently, the ionization efficacy. Short jets, which are required for maximum ion yield, were observed for solvents with relative permittivity ε_r_ higher than 25. Solvents exhibiting lower relative permittivity required the addition of 20% to 60% methanol to limit the jet length and to avoid the ineffective dripping pulsation. The observed effects were compared to conventional electrospray ionization and paper spray ionization.

## 1. Introduction

### 1.1. Discovery of Electrospray

Due to its soft ionization characteristics, electrospray has emerged as a widely used technique for the ionization of small organic compounds up to large biomolecules. The evolution of electrospray has taken place over the last century. Fundamentals were already discovered in 1913 by John Zeleny in his essential work about electric discharge from liquid points [1]. Sir Geoffrey Ingram Taylor continued to make significant contributions describing processes of liquids in a strong electric field, including the Taylor–Melcher model [2,3]. As of then, the liquid cone emerging from a spray capillary under the influence of a strong electric field became known as the Taylor cone. At the apex of the Taylor cone, the formation of a jet can be observed. The structure of the jet differs in length and diameter [4,5]. Malcolm Dole was the first to demonstrate the use of electrospray for the generation of macroions and their detection by a Faraday cage [6]. Further progress was reported by John Bennet Fenn, who demonstrated the potential of electrospray ionization to access large, labile, and nonvolatile compounds, which was not realizable by other methods [7]. This work eventually peaked in the ionization of large biomolecules by electrospray ionization coupled to mass spectrometry [8]. For his pioneering work, John B. Fenn was awarded the Nobel prize in chemistry in 2002. A comparable advancement that enabled “electrostatic atomization of liquid analytes at ambient gas pressure”, termed ERIAD, was developed by the research group of Lidija Gall. ERIAD was first used to demonstrate the ionization of progesterone and amino acids at the onset of the 1980s [9].

### 1.2. Principles of Electrospray

Despite thousands of publications per year involving electrospray ionization, only few focus on the fundamental mechanisms of ion generation by electrospray. Principles involve complex fluid dynamics and electrostatic processes, and many aspects are still the subject of discussion as emphasized by the literature [5,10,11]. A Taylor cone results from a liquid drop deformation into a conical shape in the presence of a strong electric field, typically at the tip of a thin electrically conductive capillary. For an ideal conducting liquid, a Taylor cone half angle of 49.3° was calculated, although various factors including pressure and space charge were not considered [2,5]. Numerous solvent properties, such as the relative permittivity, viscosity, surface tension, conductivity, density, and the composition of the surrounding gas impact Taylor cone formation. Moreover, the system is influenced by the applied electric potential, solvent pressure, capillary diameter, and the distance of the capillary tip to the counter electrode [5]. Recent studies even give evidence for altered solvent properties in high electric fields, such as the viscosity of water in an electric field between 0 and 0.9 V/nm, which was communicated to become unisotropic [12], and the surface tension of various liquids, which was reported to be influenced by high electric fields as well [13].

Viscosity, electrical resistivity, and solvent flow rate are described in the literature as decisive factors governing the length of the jet [4,14]. In addition, the Taylor cone angle is directly related to the applied electric potential with increasing potential resulting in a larger cone angle [15]. Taylor cones of different shapes and modes have been defined, such as tilted jets, multi jets, ramified jets, rotated jets, and pulsation modes, such as the microdripping and spindle modes [4,16,17,18,19,20,21,22]. However, the cone-jet mode is the most desired in electrospray ionization mass spectrometry, as it is mandatory for the proper ionization of analytes [18]. The jet formed at the apex of the Taylor cone breaks up and turns into a spray plume consisting of charged droplets [5,23]. In the spray plume, the ionization of analyte molecules takes place. Models of the ionization process are categorized into the ion evaporation model (IEM), charge residue model (CRM), or the chain ejection model (CEM) [10,24]. In addition, the solvent also plays a crucial role, as it directs the charge states of the analytes [25,26]. Nevertheless, further investigation of the physical effects associated with Taylor cone formation is required for gaining deeper insight into electrospray-based ionization techniques.

### 1.3. Electrospray Ionization in Ambient Mass Spectrometry

More recent developments in mass spectrometry involve ambient ionization methods, which enable direct in situ ionization without the requirement for sample preparation steps. Among them are electrospray-based methods, such as desorption electrospray ionization (DESI) [27], established in 2004, and paper spray, which was first described in 2010 [28]. Swab spray is a further electrospray-based ambient ionization method that has been introduced in 2015 by the group of Graham Cooks [29]. Meanwhile, swab spray mass spectrometry has demonstrated its potential as a hassle-free sampling and sensitive analytical method, especially for medical diagnostics, forensic investigations, and environmental analyses [30,31,32,33,34,35,36,37,38,39].

Current–voltage curves were previously applied to differentiate between the electrospray mode and the occurrence of corona discharge when spherical sampler probes were used as spray emitters [40]. The present work focuses on fundamental aspects of ion generation by swab spray with an emphasis on the influence of different solvent properties on the formation of the Taylor cone and the associated jet. Photographic observations provided evidence to differentiate between various electrospray modes and to determine the jet length, even in the absence of a signal. In the performed experiments, it was observed that the emission of a short jet is pivotal for optimizing signal yield in swab spray mass spectrometry. To evaluate ionization efficacy, a suppression marker, either salicylanilide or tetrabutylammonium iodide, was directly added to the spray solvent, and full-scan data were acquired. To our knowledge, the impact of the solvents relative permittivity on jet shaping was not demonstrated previously in ambient ionization techniques.

## 2. Results and Discussion

### 2.1. Impact of the Electric Potential on Taylor Cone Formation

The formation of a Taylor cone at the tip of a swab head can immediately be observed after the application of solvent and sufficient electric potential. Application of a potential below the onset value leads to a dripping pulsation mode, whereas the pulsation frequency increases with increasing potential. When the onset voltage is reached, the formation of a stable Taylor cone can be observed. The required onset potential depends on the type of solvent and the distance between the swab head and the counter electrode, which was held constant at 7 mm for all experiments. Manufacturing variations of swab heads can cause deviations of the onset voltage of about ±0.2 kV. Nevertheless, for methanol, ethanol, and acetone, onset voltages in the range between 3.5 and 3.9 kV were determined. The surface tensions of methanol, ethanol, and acetone correspond to 22.17 mN/m, 21.91 mN/m, and 22.71 mN/m, while the relative permittivities are 33.02, 25.32, and 21.01, respectively.

The surface tension of the liquid must be overcome by the force of the electric potential to transform the solvent droplet into a Taylor cone. Hereby, the relative permittivity (dielectric constant) acts as an insulating factor. However, a sufficient relative permittivity is necessary for charge separation, which promotes Taylor cone formation.

**Figure 1:**
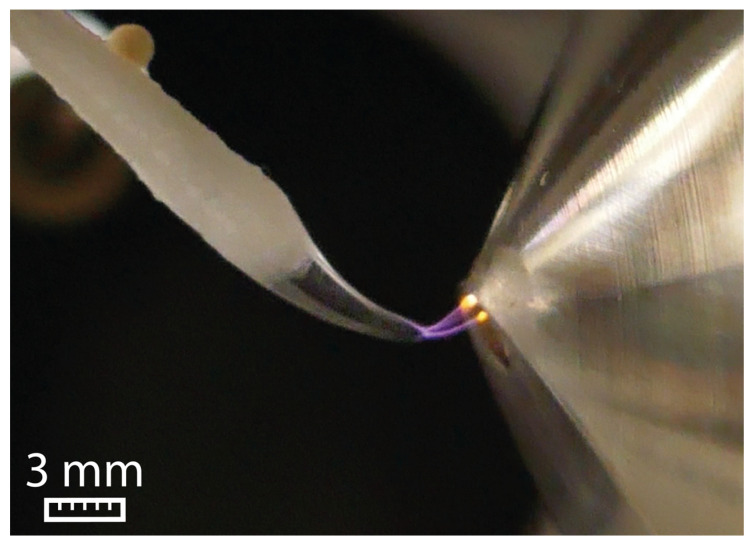
Electric discharge between the swab and the ion sweep cone serving as counter electrode for water at 5 kV and a flow rate of 45 µL/min.

The high surface tension of water (72.06 mN/m) prevents the formation of a Taylor cone. Instead, the water droplet undergoes elongation until a sudden electric discharge to the ion sweep cone takes place, as visualized in Figure 1.

Significantly higher onset potentials than found for methanol, ethanol, and acetone were observed for nitromethane (4.8 kV), dimethyl sulfoxide (DMSO) (5.3 kV), and N,N-dimethylformamide (DMF) (4.7 kV), which is explained by the higher surface tensions of these solvents. The relative permittivities of nitromethane, DMSO, and DMF are 37.27, 47.242, and 38.25, while their surface tensions are 36.53 mN/m, 42.92 mN/m, and 35.52, respectively. Toluene, which exhibits a relative permittivity of 2.38 and a surface tension of 27.91 mN/m, did not promote Taylor cone formation. The very low relative permittivity does not provide sufficient charge separation, and an addition of 50% methanol was required for Taylor cone formation at an onset potential of 4.2 kV.

**Figure 2:**
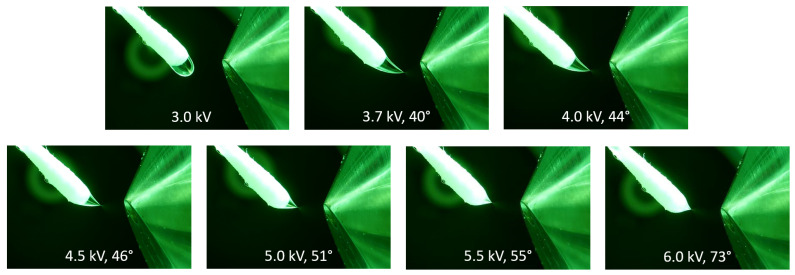
The influence of the electric potential on the Taylor cone angle at different voltages using methanol as the solvent at a flow rate of 45 µL/min. At 3.0 kV, the droplet is dripping and slightly drawn toward the counter electrode. At the onset potential of 3.7 kV, the formation of a Taylor cone becomes visible. At higher potentials, the Taylor cone angle starts to increase, which leads to a shorter cone with decreased volume.

The impact of the applied voltage on the Taylor cone angle is visualized in the series of pictures in Figure 2 using methanol as a spray solvent. The Taylor cone angle increased at elevated potentials, which was observed for all solvents that generated a short jet. Additional photographs of Taylor cones at different voltages using DMF and a mixture of toluene/methanol (50/50, *v*/*v*) is available in Supplementary Data S1. A similar relation between Taylor cone angles and applied voltages was demonstrated using spherical sampler probes [40].

### 2.2. The Different Taylor Cone Modes

The shape and stability of the Taylor cone and the emerging jet are crucial for the electrospray ionization process [5], and dripping pulsation [17,20,21,41,42] and spindle pulsation [17,18,20,21] of the Taylor cone must be avoided as well. Therefore, the applied electric potential, the solvent composition, and the flow rate of the solvent fed to the swab head must be chosen carefully. In our experiments, high ion yields were only obtained with short jets, as the ionization process takes place in the spray plume after the jet breaks up. A short jet was found to generate a voluminous spray plume which promotes efficient ionization and simultaneously facilitates the alignment of the swab in front of the electrospray interface. On the other hand, a voluminous spray plume is detrimental to accurate quantitative analysis by swab spray ionization, as part of the sprayed sample is lost at the ion sweep cone. Solvents that generate Taylor cones with long jets prevent proper ionization because the formation of a spray plume, in which the ionization process takes place, is impeded.

### 2.3. Taylor Cones with Short Jets

**Figure 3:**
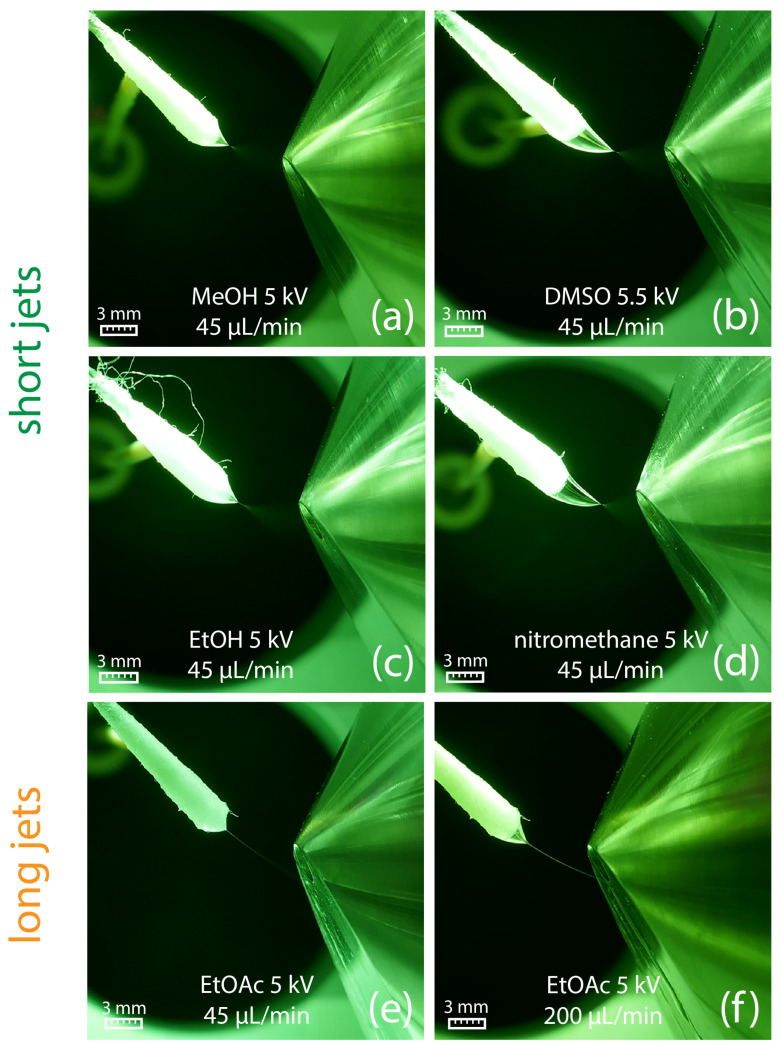
(**a**–**d**) Generation of a short jet at a flow rate of 45 µL/min for different solvents. (**e**) Ethyl acetate generates a long jet already at a solvent flow rate of 45 µL/min, which starts to break up into a spray plume in front of the counter electrode. (**f**) At a flow rate of 200 µL/min, a broader and longer jet is created, which directly hits the counter electrode.

The suitability of different solvents for efficient swab spray ionization was evaluated in a series of experiments in which a variety of solvents was tested with the aim to generate a stable Taylor cone with a short jet. Results indicated that a sufficiently high relative permittivity (ε_r_) is crucial for producing a short jet. Amongst the most suitable solvents for swab spray ionization were solvents with relative permittivity larger than 25, such as DMSO, nitromethane, acetonitrile, methanol, DMF, and ethanol (Table 1). Acetone, with a relative permittivity of 21.01, has to be considered as a borderline case, as it produced jets from short to long in multiple experiments. Ethanol, with its slightly higher relative permittivity of 25.32, always created a short jet in repeated experiments. The sprays generated by selected solvents and at different solvent flow rates are visualized in Figure 3.

**Figure 4:**
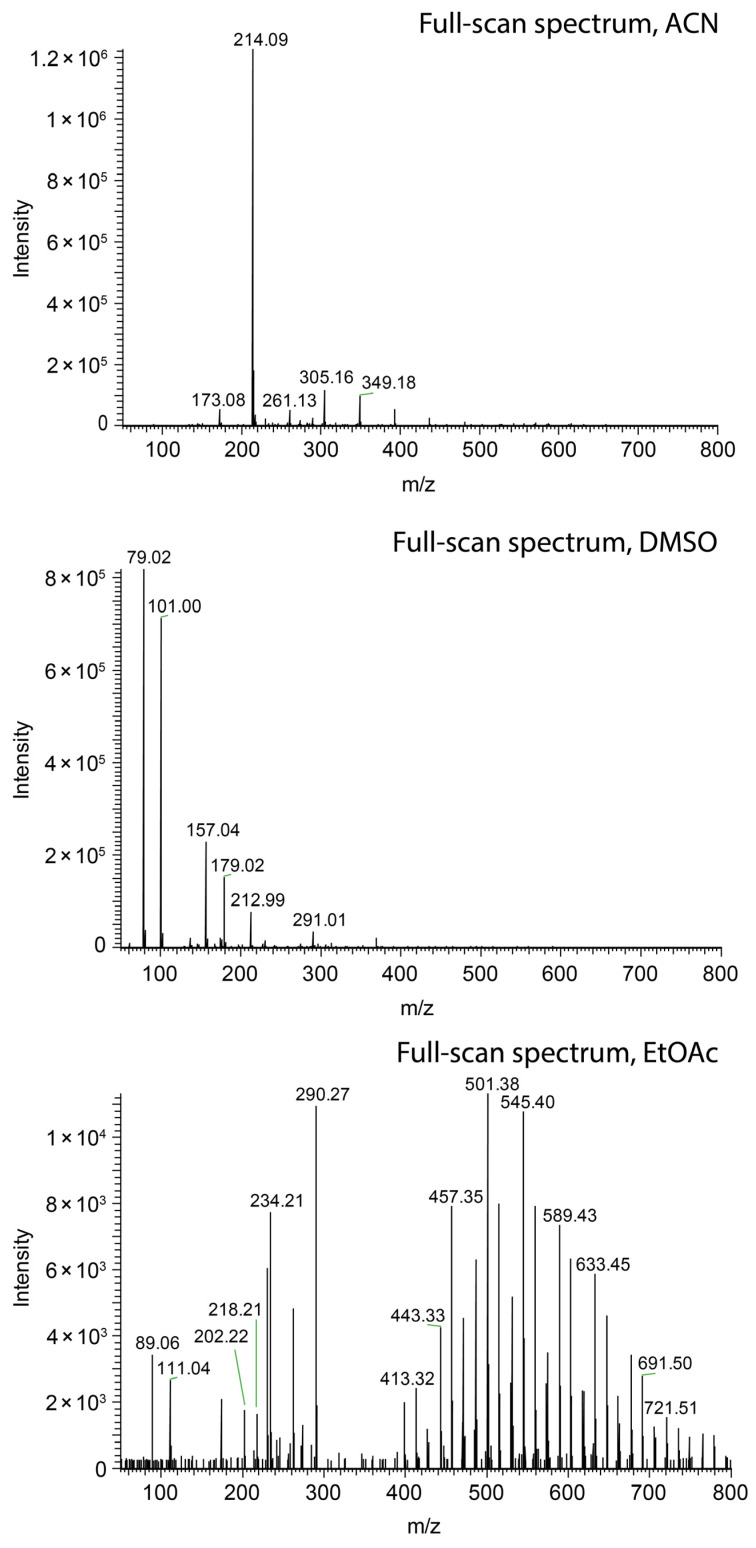
Comparison of swab spray mass spectra of salicylanilide ([M + H]^+^ at *m*/*z* 214.09) obtained with acetonitrile (**top**), DMSO (**center**), and ethyl acetate (**bottom**) as the solvents.

Nevertheless, short jets do not ensure high ionization efficacy. Methanol (Figure 3a), ethanol (Figure 3c), and acetonitrile (Figure 4, top) sprayed at 45 µL/min all resulted in the protonation of salicylanilide, which was added to the solvent at a concentration of 1 ppm for monitoring the ionization efficacy. Despite the short jet formed by DMF, DMSO, and nitromethane, no signal of the protonated salicylanilide was observed as shown for DMSO in Figure 4 (center). This is likely attributed to the low ionization efficacy of salicylanilide in these solvents. Additional full-scan spectra for several other solvents, including nitromethane, ethanol, and DMF, are available in the Supplementary Data S2.

When tetrabutylammonium iodide, a pre-charged analyte, was used as the suppression marker, comparable signal intensities were found in DMSO and DMF (spectra available in the Supplementary Data S3). This indicates that salicylanilide exhibits a lower proton affinity in these solvents.

### 2.4. Taylor Cones with Long Jets

All tested solvents with relative permittivity lower than 21 produced a long jet (see Table 1), which, in most cases, reached the ion sweep cone serving as the counter electrode in our experiments. The formation of long jets adversely affects the signal intensity, as illustrated in Figure 4 (bottom) for ethyl acetate. In addition to the solvents’ relative permittivity, the jet length is also influenced by the solvent flow rate, with higher flow rates resulting in elongated stabilized jets, as shown for ethyl acetate at 45 µL/min and 200 µL/min in Figure 3e,f, respectively. No electric discharge occurred, which was presumably due to the low electric conductivity of the ethyl acetate.

Upon spray formation, a small expansion of the swab head due to solvent saturation was observed. Therefore, the jets of solvents exhibiting a low relative permittivity, e.g., tetrahydrofuran and 2-pentanone, were not yet stabilized, and jets only appeared to be short during spray initiation but quickly expanded into longer jets after 10–30 s.

Raising the relative permittivity of the solution by the addition of methanol [45] was found to be a very effective method for jet shortening and improving the signal intensity. Table 1 shows the required portion of methanol in binary mixtures with different solvents for establishing a short jet. The methanol content was gradually increased by steps of 10% until a short jet was observed. For example, an ethyl acetate/methanol mixture in a 70/30 (*v*/*v*) ratio was required for the best results. Despite the existence of several equations to predict the relative permittivity of a mixture [46], calculations could not predict the necessary methanol percentage required to generate a short jet in solvent mixtures. We assume that the complex fluid dynamics within the Taylor cone [5] under the influence of the electric field contribute decisively to the jet formation in solvent mixtures.

In addition to methanol, a similar effect was observed by the addition of other solvents exhibiting high relative permittivity. A minimum percentage of 20% DMF or 30% nitromethane had to be mixed with ethyl acetate to obtain short jets. However, in these experiments, the resulting ion current obtained for salicylanilide was very low again.

Setting the correct solvent flow is crucial for establishing a continuous electrospray with high ionization efficacy. In favor of the ionization efficacy, the formation of small primary droplets is mandatory. Excess solvent flow results in a broader jet and larger primary droplets and eventually decreases the ionization efficacy [47]. As jet broadening comes along with jet elongation, spray plume formation comes in close proximity to the counter electrode, which is detrimental for high ion yields (Figure 3f). On the other hand, too low flow rates promote fiber spray instead of establishing a cone jet [37]. In fiber spray, the ions are emitted from a single fiber rather than from the entire swab head. Although fiber spray experiments generated a strong analyte signal with low background, we experienced low reproducibility and inefficient analyte extraction from the swab, which impedes quantitative work [39]. Consequently, the aim is to set the flow rate as low as possible without disrupting the electrospray and simultaneously tolerating the positional and geometrical variations of the swab heads.

### 2.5. Dripping Pulsation Mode

**Figure 5:**
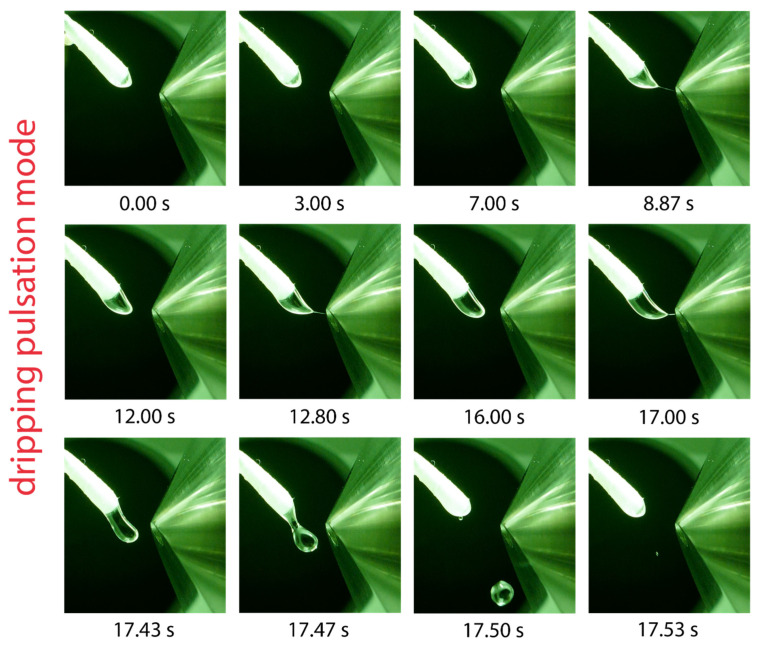
Visualization of one dripping pulsation cycle using toluene as the solvent, a flow rate of 45 µL/min and a potential of 5.5 kV applied to the swab. Droplet elongation and sporadically occurring jet formation is visible before the elongated droplet is ejected after 17.5 s and another cycle starts.

Solvents with the lowest relative permittivities examined in this work include hexane, 1,4-dioxane, toluene, and dibutyl ether. In all cases, the formation of a pulsating Taylor cone was observed. The pulsation mode of toluene is exemplarily shown in the series of photographs in Figure 5. The solvent droplet emerging from the swab head undergoes a slow elongation process and approaches the counter electrode until the droplet is suddenly ejected after 17.47 s. During the elongation process, the formation of a tiny jet is shortly visible, which can be observed at 8.87, 12.80, and 17.00 s in Figure 5; however, no ions were generated. The pulsation frequency was found to depend on the applied voltage and flow rate; increasing the voltage and flow rate led to a higher pulsation frequency. This kind of pulsation is referred to as the “dripping pulsation mode”, as reported previously for capillary-based electrospray [17,20,21,41,42]. The solvent must allow electric field-induced charge separation to generate a Taylor cone emitting a spray of charged primary droplets [48]. Strongly polar solvents that exhibit a high relative permittivity support charge separation in the Taylor cone. The lack of charge separation in solvents with very low relative permittivity inhibits Taylor cone formation and is likely to be responsible for the dripping pulsation mode.

To cure the dripping pulsation mode problem, the addition of methanol proved to be successful. For example, toluene mixed with 50% methanol generated a stable spray with a short jet (visualization is provided in Supplementary Data S1). Furthermore, the mixture of toluene/methanol (50/50, *v*/*v*) provided abundant ionization efficacy for both salicylanilide and tetrabutylammonium iodide as suppression markers (full-scan spectra are presented in Supplementary Data S2 and S3).

### 2.6. Spindle Pulsation Mode

The pulsation mode observed for solvents with a very low relative permittivity differs from the pulsation modes observed for polar solvents. Although methanol mostly produced a stable Taylor cone with a short jet, pulsation was observed in certain cases, especially at elevated flow rates or when the swab position or applied potential were changed during operation. In rare cases, pulsation shortly occurred at initiation of the spray or randomly during operation.

**Figure 6:**
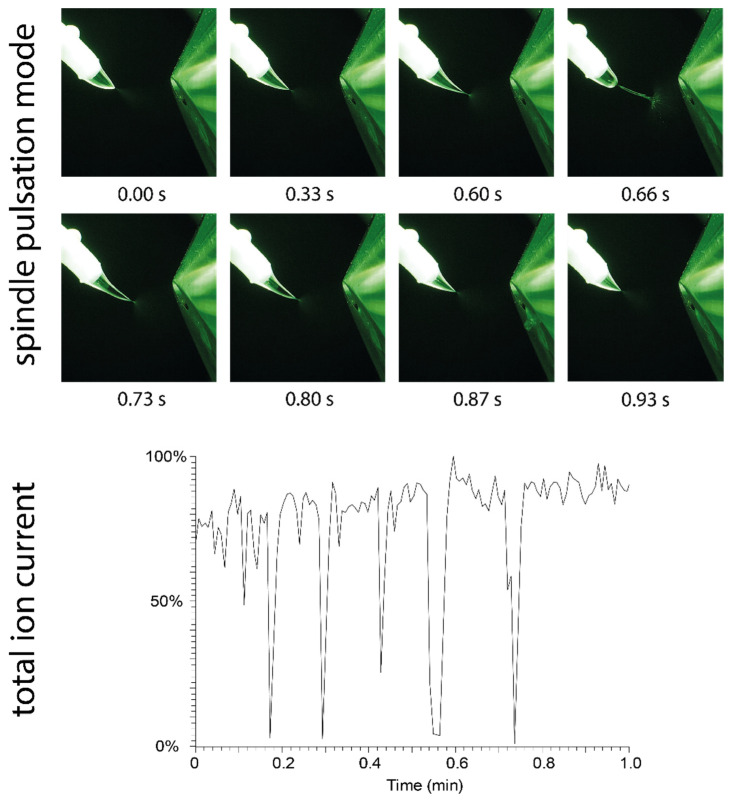
(**Top**): Visualization of the spindle mode: The Taylor cone formed by methanol undergoes an elongation process, which creates a spindle-shaped droplet that is quickly ejected after 0.66 s. (**Bottom**): The impact of repeated spindle mode pulsation events on the total ion current is shown. During ejection of the spindle, the spray plume collapses, and no ions are generated.

The pulsation mode of methanol, which was easily provoked by increasing the distance to the counter electrode during operation (from 7 mm to approximately 9 mm), is shown in Figure 6. This pulsation mode is referred to as the “spindle mode” [17,18,20,21], which generates fluctuating ion currents. The broadening and elongation of the jet eventually leads to the ejection of a spindle-shaped droplet. Due to ejection of the droplet, the spray plume collapses and ion generation ceases. Figure 6 shows the total ion current chromatogram recorded under spindle mode conditions. The size of the ejected droplets varies, and breakdown of the ion current occurs within irregular cycles of several seconds under the given conditions (45 µL/min solvent flow and 5.5 kV potential).

### 2.7. Taylor Cones with Long Jets by Non-Viscous Solvents

The deposition of pure highly viscous glycerol (η = 934 mPa·s) to a Copan 160C swab resulted in a drastic increase in the jet length, which is in agreement with the common understanding that the jet length is directly related to the viscosity of the solvent due to better stabilization [4,14]. However, our experiments revealed that the jet lengths of organic solvents suitable for electrospray ionization do not follow the order of viscosities. Investigated solvents span over a viscosity range of approximately 0.2 to 2.0 mPa·s. The viscosities of solvents are given in Table 1, and the observed jet characteristics are indicated by the font color. Notably, diethyl ether, which has the lowest viscosity, produced a long jet. This stands in contrast to DMSO, which exhibits the second highest viscosity among the listed solvents and produced a short jet. The higher relative permittivity of DMSO promotes charge separation within the jet under the influence of the electric field. The resulting higher charge density helps to overcome the surface tension, facilitating jet break-up into the plume of charged droplets, which eventually leads to ionization. The opposite process applies to the production of a long jet by diethyl ether. Therefore, the long jets formed by organic solvents with low viscosity cannot be the result of spray stabilization by viscosity but are related to their low relative permittivity. Overcoming the surface tension by the electric force is required for spray plume formation and subsequent ionization [49,50,51]. Low relative permittivity dampens charge separation within the jet and consequently prevents spray plume formation and ionization.

**Figure 7:**
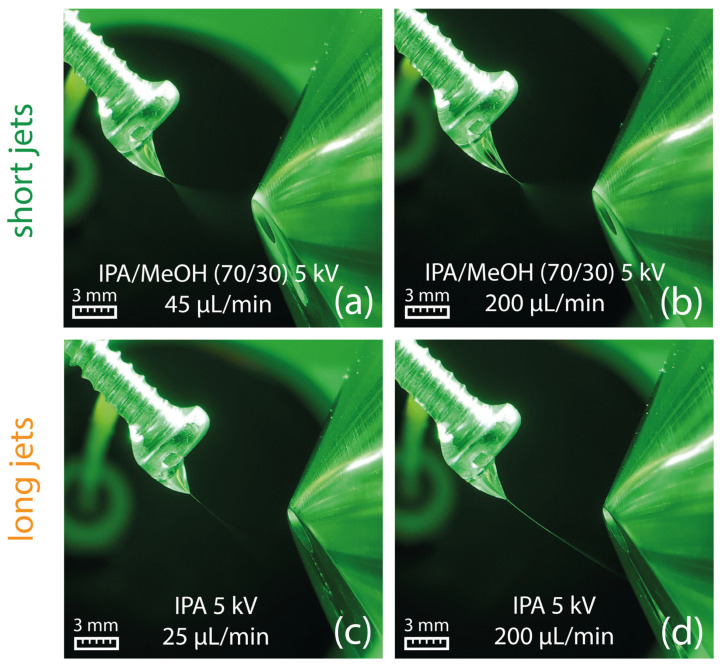
Formation of a Taylor cone with a short jet using 2-propanol/methanol (70/30, *v*/*v*) at 45 µL/min (**a**) and 200 µL/min (**b**). Formation of a Taylor cone with a long jet on a metal screw using 2-propanol at a flow rate of 25 µL/min (**c**) and 200 µL/min (**d**).

Concerns that solvents of lower polarity may extract adhesives and other swab-related contaminants, which could potentially influence solvent properties such as the viscosity, were refuted by an experiment, where the swab was replaced by a metal screw. The metal screw imitates the swab by offering solvent accumulation on the screw head similar to a swab head but without the risk of adhesive extraction. The sprays generated by 2-propanol and a mixture of 2-propanol (70/30, *v*/*v*) with methanol from a metal screw are shown in Figure 7, demonstrating that the flow-related characteristic of the Taylor cone is comparable to the experiments with Copan 160C swabs shown in Figure 3e,f. Consequently, a contribution from the swab material can be excluded.

Although recent studies revealed that properties of liquids are altered in strong electric fields, the influence on electrospray ionization has not been investigated yet. Available data indicate only minor changes in very high electric fields [12,13]. Therefore, the use of standard solvent constants is considered appropriate to explain the phenomenon of increasing jet length. Conclusively, for the investigated solvents, the relative permittivity of solvents measured under standard condition is the decisive factor when it comes to jet shaping.

### 2.8. Jet Formation in Capillary-Based Electrospray Ion Sources

**Figure 8:**
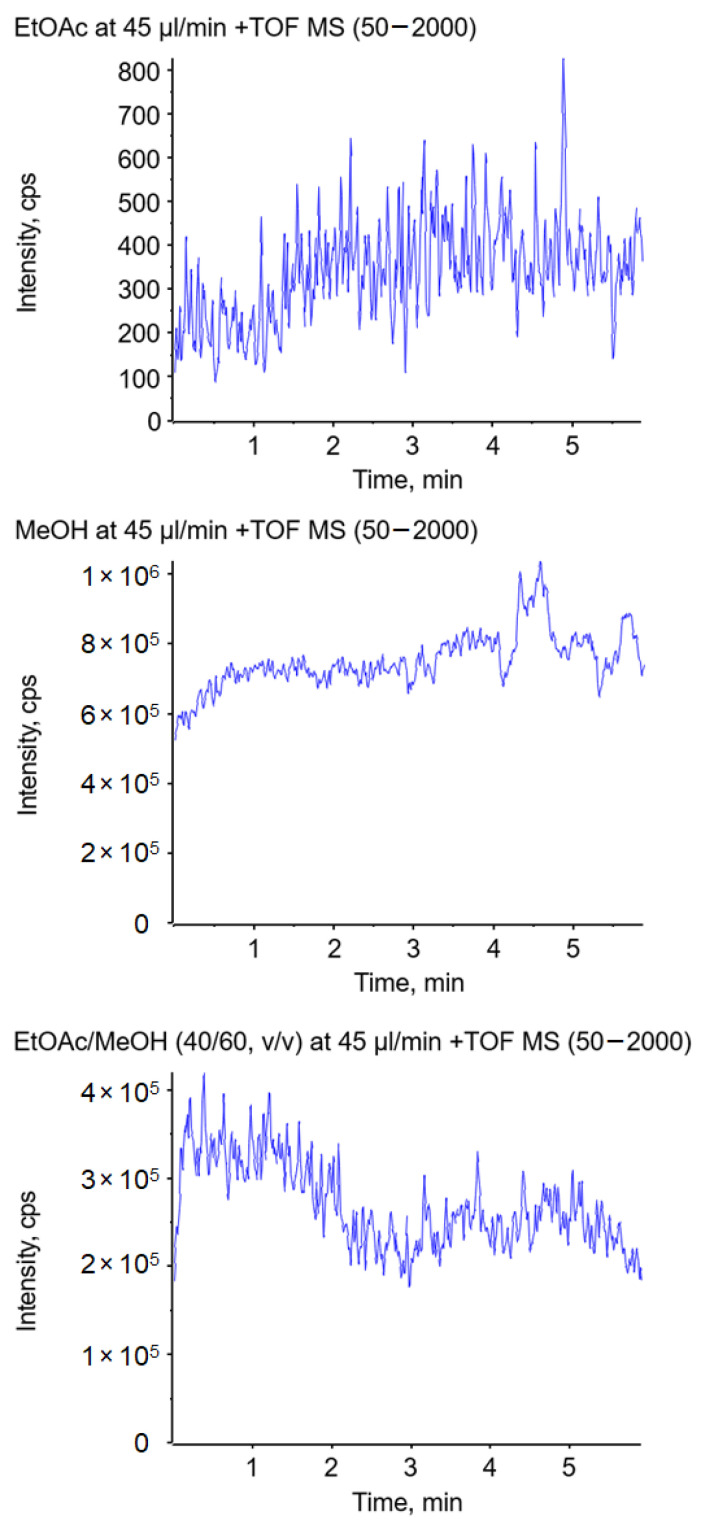
Impact of the solvent on the total ion current using ethyl acetate (**top**), methanol (**center**), and a mixture of ethyl acetate/methanol (40/60, *v*/*v*) (**bottom**) in a conventional electrospray ion source without the assistance of nebulizer and heater gas.

To further investigate the described effects, a similar experiment was performed in a conventional electrospray ion source. Hereby, the solvent was pumped through a thin conductive electrospray emitter. The nebulizer and heater gas were turned off to prevent disintegration of the jet by gas flow. The solvent, including 0.1% formic acid and 1 ppm suppression marker, was delivered at a flow rate of 45 µL/min in all experiments. The use of ethyl acetate as the spray solvent delivered hardly any signal over a period of five minutes, as illustrated in Figure 8 (top), which must be related to the formation of a long jet. When methanol was used as the spray solvent, an abundant signal over the course of five minutes could be observed (Figure 8 center). A significant improvement of the ionization was achieved by the addition of methanol to ethyl acetate, resulting in ion currents comparable to the ones obtained with pure methanol (shown in Figure 8 (bottom) for 60% MeOH). Compared to the swab spray experiments, the higher percentage of MeOH is most likely due to the different electrospray emitter geometry.

These experiments demonstrate that the observed effect of the relative permittivity on jet formation is transferable to other electrospray ion sources that do not provide ion source gases and are operated at comparably low solvent flow rates. For ambient ionization that does not provide gas to assist ionization, the relative permittivity of the spray solvent is a crucial factor, as the formation of a short jet is mandatory for ionization.

### 2.9. Jet Formation in Paper Spray

The formation of a short jet is crucial for efficient ion generation in any electrospray-based ionization technique. In swab spray ionization, the Taylor cone and the subsequent jet emit from a rather large and geometrically less defined surface area, which is opposed to the conventional well-defined capillary emitters. In paper spray, the sample is deposited on a triangular piece of paper, and the spray is formed at the vertex pointing toward the counter electrode. Compared to swab spray, the Taylor cone originates from a smaller surface area, and spray emission is supported by paper fibers. Indeed, paper spray requires lower ionization potential and a reduced solvent flow rate [52,53,54]. In our experiments, a potential of +3.5 kV and a flow rate of 30 μL/min only were sufficient to generate a stable electrospray in our experiments using different solvents.

**Figure 9:**
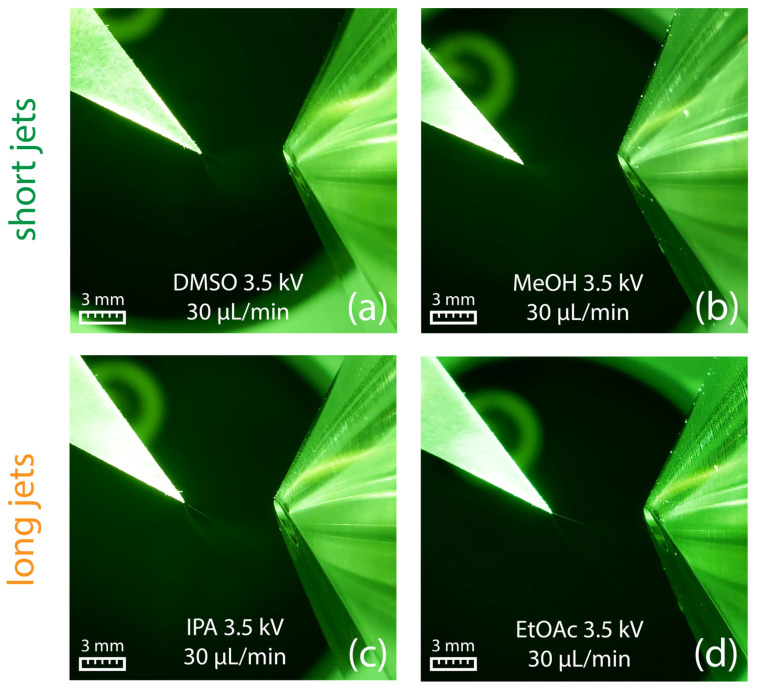
Paper spray in combination with DMSO (**a**) and methanol (**b**) produced short jets. In contrast, 2-propanol (**c**) and ethyl acetate (**d**) led to the formation of long jets. All pictures were obtained using a flow rate of 30 µL/min in combination with an electric potential of +3.5 kV.

Similar to swab spray, DMSO and methanol resulted in short jets (Figure 9a,b), whereas longer jets were observed for 2-propanol and ethyl acetate (Figure 9c,d). Under the given conditions, paper spray was found to be not susceptible to the adverse spindle pulsation mode observed in swab spray, which can be attributed to paper fibers that support spray emission.

**Figure 10:**
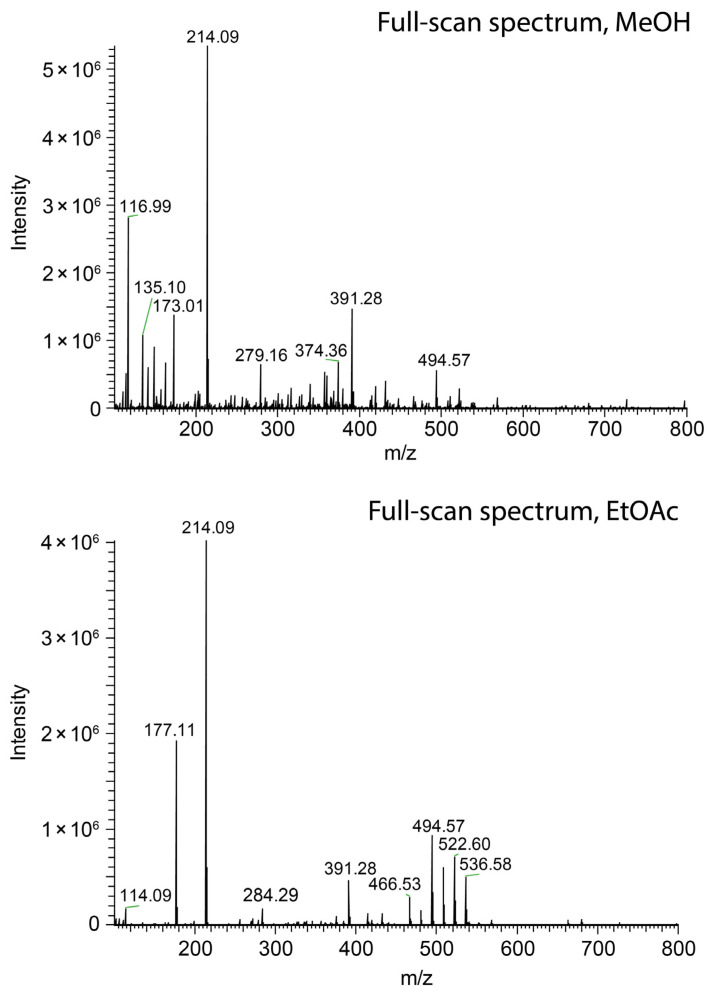
Comparison of paper spray mass spectra of salicylanilide ([M + H]^+^ at *m*/*z* 214.09) obtained with methanol (**top**) and ethyl acetate (**bottom**) as the solvents.

The ionization efficacy of paper spray was probed by monitoring the ion current of salicylanilide, which was added to the spray solvent at a concentration of 1 ppm. As in the case of swab spray, jet-shaping effects governed by the solvent relative permittivity were observed. However, the detrimental effect on ion generation was not encountered. Ethyl acetate as the solvent yielded an ion current of protonated salicylanilide comparable to the one detected with methanol, as indicated by the peaks at *m*/*z* 214.09 in Figure 10. The loss of solvent by evaporation and the occurrence of multiple jets, as evidenced by Figure 9a,c,d, further reduced the effective flow rate in individual jets. These effects counteract the formation of very long jets and enable ionization even in the case of 2-propanol and ethyl acetate as the solvents. Hence, paper spray is less sensitive to solvent selection and does not require the same level of solvent optimization as swab spray.

## 3. Material and Methods

### 3.1. Instrumentation

The instrumentation consisted of a custom-made swab spray ion source connected to a Thermo Fisher Scientific (Reinach, Switzerland) LTQ Orbitrap Velos mass spectrometer. The ion source allowed swab fixation in a 45-degree angle, direct application of the electric potential, and continuous solvent supply by a syringe pump. The method has previously been described in detail [39]. All experiments were performed in positive ionization mode. For Taylor cone formation, the solvent flow rate was set to 45 µL/min (with the exception of acetone, dichloromethane, and diethyl ether, where the flow rate was set to 60 µL/min due to high solvent evaporation rates). The solvent flow was started about 1–2 min prior to the application of the electric potential to ensure solvent saturation of the swab head. A distance of 7 mm from the swab head to the counter electrode was chosen for all experiments, as such a setup was beneficial for signal intensity and for the prevention of electrical discharges. For photographic observation, a green flashlight was placed on top of the ion source housing (replacing the top-mounted camera), whereas the pictures were taken from the side. To monitor the ionization efficacy and background signal, salicylanilide was added to all solvents as a suppression marker at a concentration of 1 ppm. To facilitate ionization, 0.1% formic acid was added to all solvent combinations.

A Turbo V ion source was used for conventional electrospray on a Sciex X500B (AB Sciex, Baden, Switzerland) quadrupole time-of-flight mass spectrometer operated in the positive ionization mode at +5.5 kV. The curtain gas was set to 35 psi, and the solvent was supplied at a flow rate of 45 µL/min. The nebulizer and heater gas were turned off in these experiments.

### 3.2. Chemicals and Materials

The following chemicals were purchased from Merck/Sigma-Aldrich (Buchs, Switzerland): methanol, ethyl acetate, acetonitrile, 2-propanol (all Supelco Lichrosolv^®^ hypergrade for LC-MS, Merck/Sigma-Aldrich (Buchs, Switzerland)), trichloromethane (Chromasolv™ plus for HPLC, 99.8%, Honeywell Specialty Chemicals (Seelze, Germany)), formic acid (for LC-MS, 98–100%), water (LC-MS grade), toluene (HPLC grade, 99.9%), methyl acetate (99.5%, anhydrous), 3-pentanone (≥99.0%), propyl acetate (99%), dibutyl ether (≥99.0%), 1-nitropropane (≥98.5%), DMSO (≥99.0%), n-hexane (≥99%), 1,4-dioxane (≥99%), N,N-dimethylformamide (≥99.8%), anisole (99%), tetrabutylammonium iodide (98%), and salicylanilide (99%). Nitromethane (≥98%) and dichloromethane (99.7%, HPLC grade, stabilized with amylene) were purchased from Thermo Fisher Scientific (Reinach, Switzerland). THF and acetone (both HPLC grade, HiPerSolv Chromanorm^®^, VWR International (Dietikon, Switzerland)) were both purchased from VWR International (Dietikon, Switzerland). Ethanol absolute (HPLC grade) was purchased from Honeywell Specialty Chemicals (Seelze, Germany) and 1-chlorobutane (biopure solvent) was purchased from Romil (Waterbeach, UK).

The Copan 160C minitip rayon swabs with aluminum applicator contained in a sealed plastic tube were purchased from VWR International (Dietikon, Switzerland). A swab prewashing procedure was applied by ultrasonication in ethyl acetate according to the literature [39]. The metal screw which was used as an electrospray emitter was cleaned by ultrasonication in 2-propanol for 15 min at 40 °C. For paper spray, filter paper type 589/1 (Schleicher & Schuell, Göttingen, Germany) was cut into a triangle and mounted in the swab holder at a distance of approximately 7 mm from the counter electrode. The solvent supply capillary was directly positioned on the upper part of the filter paper.

## 4. Conclusions

The formation of a Taylor cone with a short spray jet is mandatory for successful electrospray-based swab spray mass spectrometry. The present investigation shows that the relative permittivity of spray solvents is the key parameter in swab spray that determines the electrospray mode and therefore the ionization efficacy. Solvents are subdivided into three groups that exhibit different electrospray modes. Solvents with a relative permittivity larger than 25 were found to generate a Taylor cone with a short jet that enables high ionization efficacy, whereas spray solvents with intermediate relative permittivity in the range between 4 and 25 formed Taylor cones with long jets resulting in decreased ionization efficacy. A dripping pulsation mode was observed for solvents with low relative permittivity below 4. The addition of varying portions of methanol is recommended or even required to raise the relative permittivity of spray solvents that exhibit long jets or a dripping pulsation mode. On the other hand, ionization efficacy in paper spray remained unaffected by elongated jets due to different spray emission and lower flow rates.

## Figures and Tables

**Table 1:** Relative permittivity, viscosity, and surface tension of various pure solvents [43,44]. The green-colored solvents allow the formation of a Taylor cone with a short jet. The yellow-colored solvents lead to the formation of long jets, whereas the red-colored solvents result in a dripping pulsation mode. The required portion of methanol to produce a short jet is given in volume percentage. Water is in yellow color, as it represents a special case, where strong surface tension was preventing Taylor cone formation. Glycerol is in brown color, because its high viscosity produced a long jet. Full-scan data of various solvent mixtures with methanol are provided in Supplementary Data S2.

Spray Mode	Solvent	MeOH Content *v*/*v* in % for Short Jet	Relative Permittivity ε_r_ (20° C)	Viscosity η (25 °C) mPa•s	Surface Tension γ (25 °C) mN/m
**dripping pulsation**	hexane	immiscible	1.887	0.300	17.88
	1,4-dioxane	≥60	2.219	1.177	32.75
	toluene	≥50	2.379 (23 °C)	0.560	27.91
	dibutyl ether	≥60	3.083	0.637	N/A
**Taylor cone with long jet**	diethyl ether	≥30	4.267	0.224	16.50
	anisole	≥50	4.302 (21 °C)	1.056	35.10
	trichloromethane	≥20	4.807	0.537	26.65
	propyl acetate	≥30	5.622	0.544	23.80
	ethyl acetate	≥30	6.081	0.423	23.39
	methyl acetate	≥30	7.072 (15 °C)	0.364	24.73
	1-chlorobutane	≥40	7.276	0.422	23.18
	tetrahydrofuran	≥30	7.522 (22 °C)	0.456	26.40 (20 °C)
	dichloromethane	≥20	8.936 (25 °C)	0.413	27.20
	3-pentanone	≥30	17.002	0.444	24.74
	2-propanol	≥30	20.182	2.040	20.92
	acetone	≥20	21.012	0.306	22.71
	1-nitropropane	≥30	24.702 (15 °C)	0.798	N/A
**Taylor cone with short jet**	ethanol		25.320	1.074	21.91
	methanol		33.020	0.544	22.17
	acetonitrile		36.642	0.369	28.66
	nitromethane		37.272	0.630	36.53
	*N,N*-dimethylformamide		38.250	0.794	35.52
	dimethyl sulfoxide		47.242	1.987	42.92
	water	≥90	80.120	0.890	72.06
	glycerol		46.532	934	62.50

## Data Availability

Additional data is provided in the Supplementary Materials. Further data is available upon request.

## Supplementary Materials

The following supporting information can be downloaded at: https://www.mdpi.com/article/10.3390/molecules29174274/s1, Figure S1: Taylor cone angles at different voltages using toluene/methanol (50/50, *v*/*v*) and dimethyl formamide; Figure S2: Full-scan spectra of nitromethane and ethanol; Figure S3: Full-scan spectra of trichloromethane/methanol (80/20, *v*/*v*), ethyl acetate/methanol (70/30, *v*/*v*), and methanol; Figure S4: Full-scan spectra of dibutyl ether/methanol (40/60, *v*/*v*), acetone/methanol (80/20, *v*/*v*), and dimethyl formamide; Figure S5: Full-scan spectra of anisole/methanol (50/50, *v*/*v*), toluene/methanol (50/50, *v*/*v*), and diethyl ether; Figure S6: Full-scan spectra of dimethyl formamide, dimethyl sulfoxide, and toluene/methanol (50/50, *v*/*v*)
