# Supplementary material for: Influence of Solvent Relative Permittivity in Swab Spray Mass Spectrometry
Source: Molecules. 2024 Sep 9;29(17):4274. doi: 10.3390/molecules29174274 (PMC11397147; doi:10.3390/molecules29174274)
Supplement: Supplementary file 1 [file molecules-29-04274-s001.zip › molecules-3158992-supplementary.pdf]

molecules MDPI

*Supplementary*

# Influence of solvent relative permittivity in swab spray mass spectrometry

**Thomas Muggli and Stefan Schürch ***

Department of Chemistry, Biochemistry and Pharmaceutical Sciences, University of Bern, 3012 Bern, Switzerland

**Supplementary Data**

S1. Taylor Cone Angles at Different Voltages:

Figure S1: Taylor cone angles at different voltages using toluene/methanol (50/50, v/v) and dimethyl formamide

S2. Full-Scan Swab Spray Spectra of Various Solvents Using Salicylanilide as Suppression Marker:

Figure S2: Full-scan spectra of nitromethane and ethanol

Figure S3: Full-scan spectra of trichloromethane/methanol (80/20, v/v), ethyl acetate/methanol (70/30, v/v), and methanol

Figure S4: Full-scan spectra of dibutyl ether/methanol (40/60, v/v), acetone/methanol (80/20, v/v), and dimethyl formamide

Figure S5: Full-scan spectra of anisole/methanol (50/50, v/v), toluene/methanol (50/50, v/v), and diethyl ether

S3. Full-Scan Swab Spray Spectra of Various Solvents Using Tetrabutylammonium Iodide as Suppression Marker:

Figure S6: Full-scan spectra of dimethyl formamide, dimethyl sulfoxide, and toluene/methanol (50/50, v/v)

**S1 Taylor Cone Angles at Different Voltages**

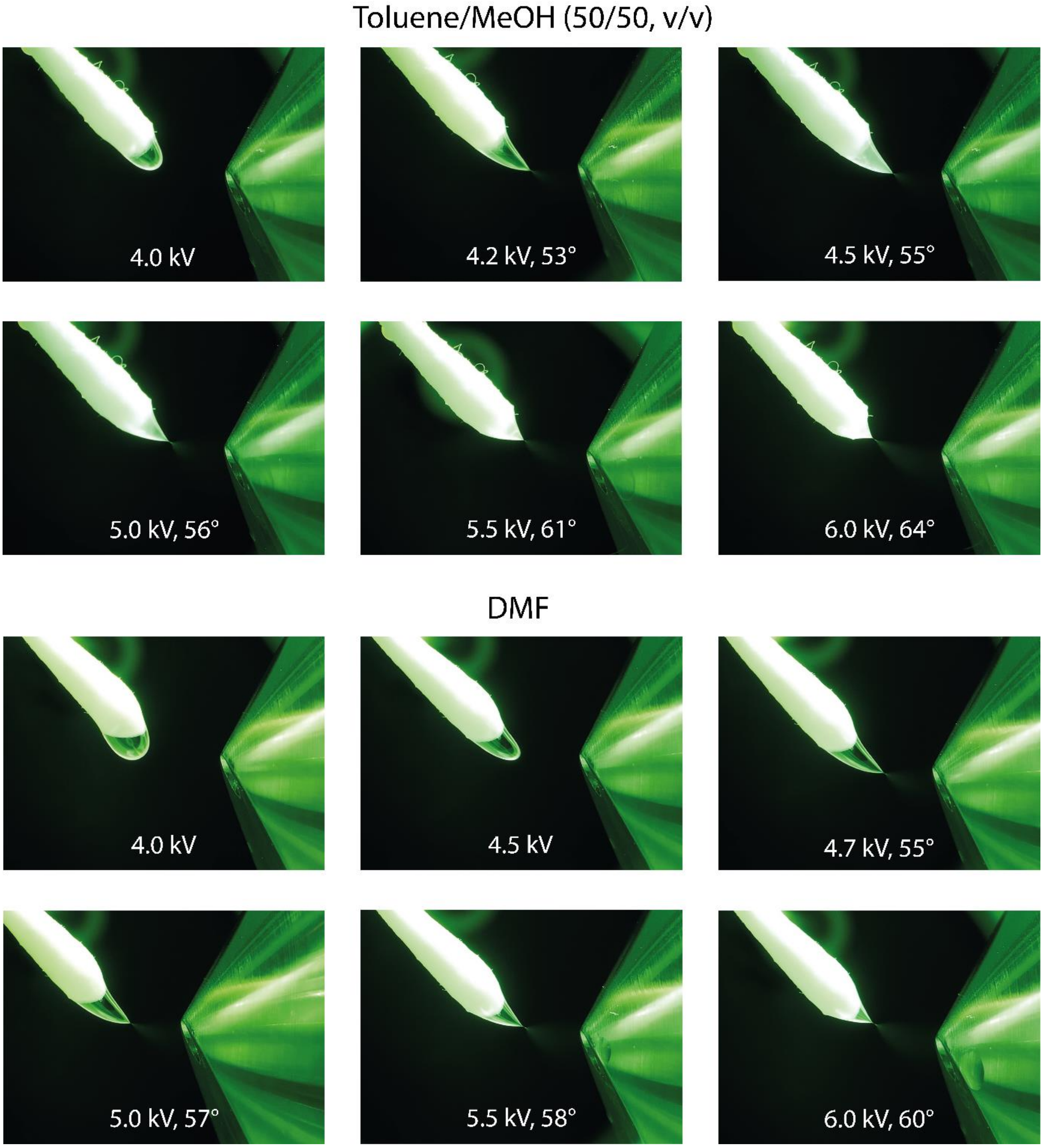


**Figure S1.** The influence of the electric potential on the Taylor cone angle at different voltages using toluene/methanol (50/50, v/v) on the top and dimethyl formamide on the bottom. Former exhibited an onset voltage of 4.2 kV and latter of 4.7 kV.

## S2. Full-Scan Swab Spray Spectra of Various Solvents Using Salicylanilide as Suppression Marker

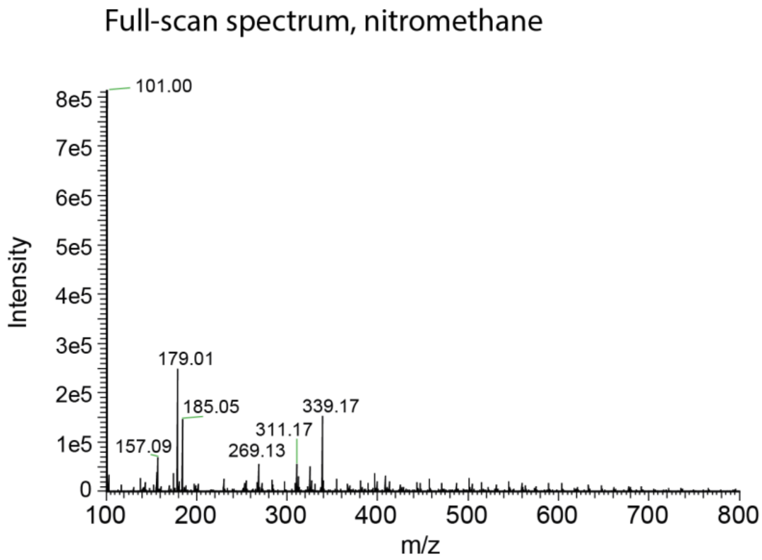


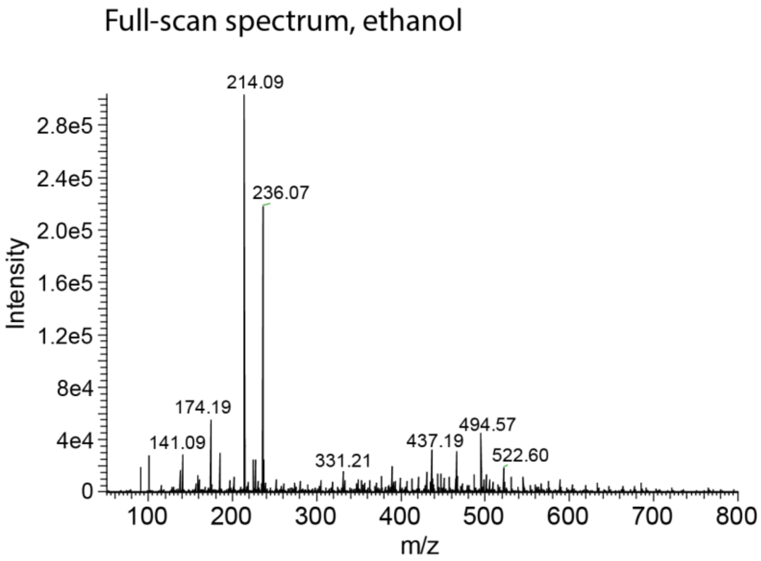


**Figure S2.** Illustration of swab spray mass spectra of salicylanilide ($[M+H]^+$ at m/z 214.09) obtained with nitromethane (top) and ethanol (bottom) as the solvents. Both solvents generated a short jet, but only ethanol enabled the ionization of the salicylanilide suppression marker.

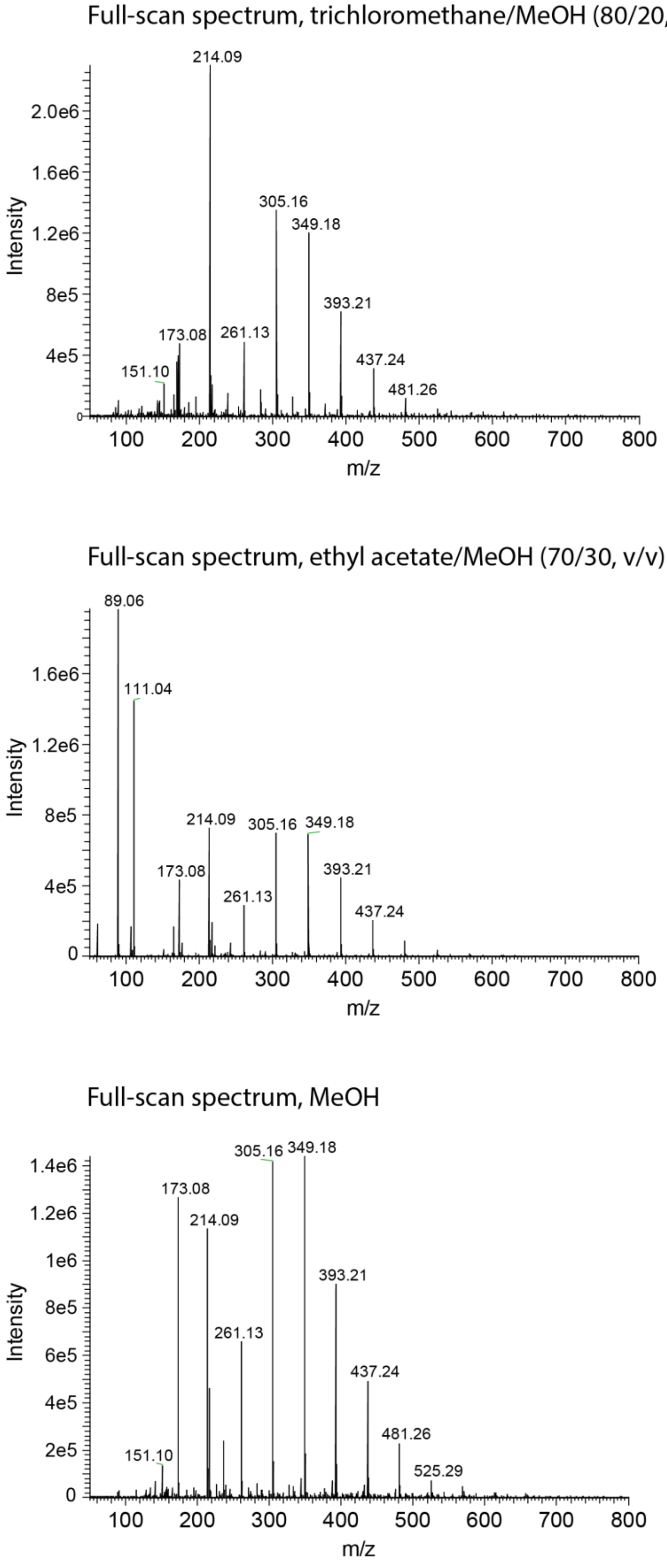


**Figure S3.** Depiction of swab spray mass spectra of salicylanilide ($[M+H]^+$ at m/z 214.09) obtained with trichloromethane/methanol (80/20, v/v) (top), ethyl acetate/methanol (70/30, v/v) (center), and methanol (bottom) as the solvents. All three solvents generated a short jet and provided sufficient ionization for the salicylanilide suppression marker.

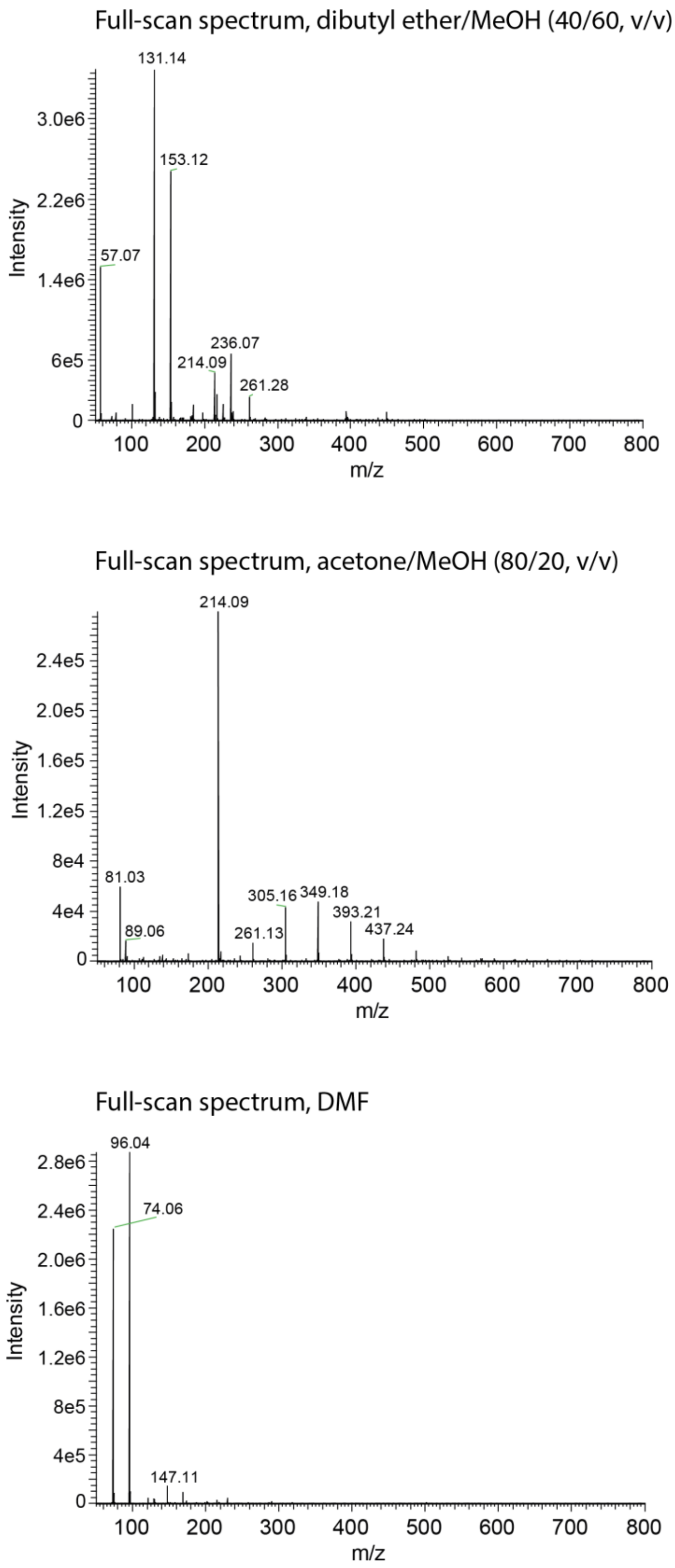


**Figure S4.** Illustration of swab spray mass spectra of salicylanilide ($[M+H]^+$ at m/z 214.09) obtained with dibutyl ether/methanol (40/60, v/v) (top), acetone/methanol (80/20, v/v) (center), and dimethyl formamide (bottom) as the solvents. Former two solvents generated a short jet and provided ionization of the suppression marker. Despite the formation of a short jet by dimethyl formamide, the ionization efficacy for the suppression marker salicylanilide was not sufficient.

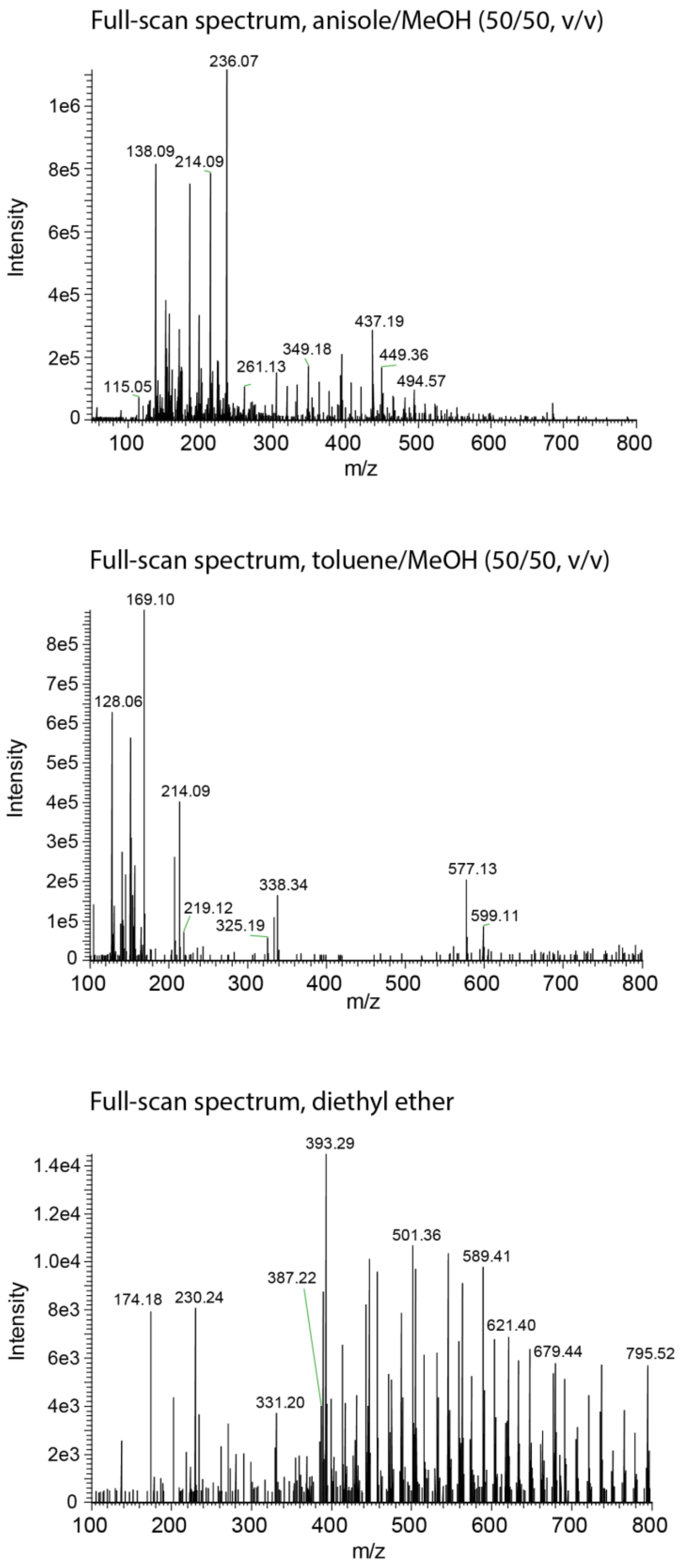


**Figure S5.** Visualization of swab spray mass spectra of salicylanilide ($[M+H]^+$ at m/z 214.09) obtained with anisole/methanol (50/50, v/v) (top), toluene/methanol (50/50, v/v) (center), and diethyl ether (bottom) as the solvents. Former two solvents generated a short jet and provided sufficient ionization for the suppression marker salicylanilide. On the contrary, diethyl ether generated an extended jet, which consequently impeded the ionization process of salicylanilide.

## S3 Full-Scan Swab Spray Spectra of Various Solvents Using Tetrabutylammonium Iodide as Suppression Marker

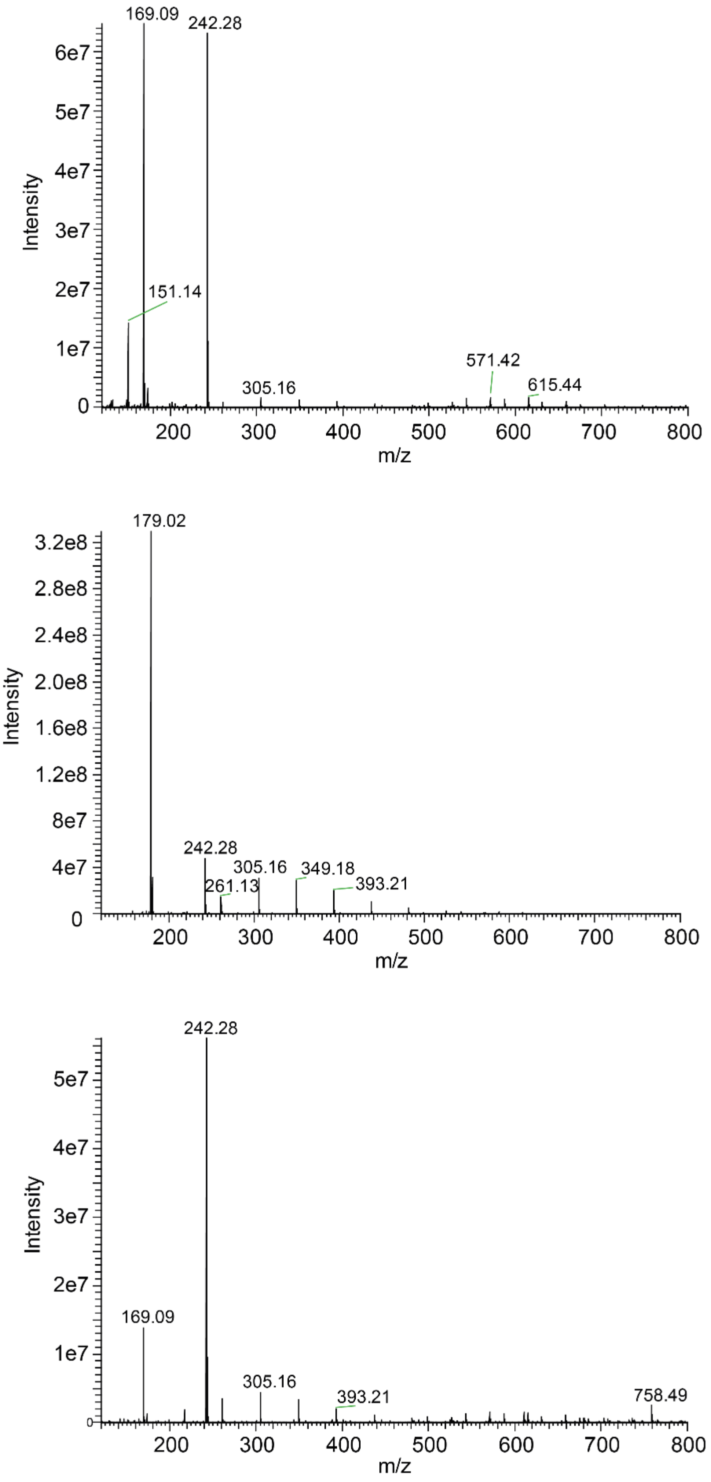


**Figure S6.** Illustration of swap spray mass spectra of tetrabutylammonium iodide ([M]$^+$ at m/z 242.28) obtained with dimethyl formamide (top), dimethyl sulfoxide (center), and toluene/methanol (50/50, v/v) (bottom). All solvents produced a short jet region and exhibited a comparable signal intensity of the suppression marker tetrabutylammonium iodide.
